# Production of Fucoxanthin from Microalgae *Isochrysis galbana* of Djibouti: Optimization, Correlation with Antioxidant Potential, and Bioinformatics Approaches

**DOI:** 10.3390/md22080358

**Published:** 2024-08-06

**Authors:** Fatouma Mohamed Abdoul-Latif, Ayoub Ainane, Laila Achenani, Ali Merito Ali, Houda Mohamed, Ahmad Ali, Pannaga Pavan Jutur, Tarik Ainane

**Affiliations:** 1 Medicinal Research Institute, Center for Research and Study of Djibouti, Djibouti City P.O. Box 486, Djibouti; 2 Superior School of Technology, University of Sultan Moulay Slimane, P.O. Box 170, Khenifra 54000, Moroccolaila.achenani@usms.ma (L.A.); 3 Peltier Hospital of Djibouti, Djibouti City P.O. Box 2123, Djibouti; 4 University Department of Life Sciences, University of Mumbai, Vidyanagari, Santacruz (East), Mumbai 400098, India; 5 Omics of Algae Group, Industrial Biotechnology, International Centre for Genetic Engineering and Biotechnology, Aruna Asaf Ali Marg, New Delhi 110067, India; pavan.jutur@icgeb.org

**Keywords:** antioxidant, carotenoids, fucoxanthin, in silico studies, microalgae, production, statistical approaches

## Abstract

Fucoxanthin, a carotenoid with remarkable antioxidant properties, has considerable potential for high-value biotechnological applications in the pharmaceutical, nutraceutical, and cosmeceutical fields. However, conventional extraction methods of this molecule from microalgae are limited in terms of cost-effectiveness. This study focused on optimizing biomass and fucoxanthin production from *Isochrysis galbana*, isolated from the coast of Tadjoura (Djibouti), by testing various culture media. The antioxidant potential of the cultures was evaluated based on the concentrations of fucoxanthin, carotenoids, and total phenols. Different nutrient formulations were tested to determine the optimal combination for a maximum biomass yield. Using the statistical methodology of principal component analysis, Walne and Guillard F/2 media were identified as the most promising, reaching a maximum fucoxanthin yield of 7.8 mg/g. Multiple regression models showed a strong correlation between antioxidant activity and the concentration of fucoxanthin produced. A thorough study of the optimization of *I. galbana* growth conditions, using a design of experiments, revealed that air flow rate and CO_2_ flow rate were the most influential factors on fucoxanthin production, reaching a value of 13.4 mg/g. Finally, to validate the antioxidant potential of fucoxanthin, an in silico analysis based on molecular docking was performed, showing that fucoxanthin interacts with antioxidant proteins (3FS1, 3L2C, and 8BBK). This research not only confirmed the positive results of *I. galbana* cultivation in terms of antioxidant activity, but also provided essential information for the optimization of fucoxanthin production, opening up promising prospects for industrial applications and future research.

## 1. Introduction

Microalgae are emerging as a game-changer in the realm of natural ingredients [1]. These microscopic organisms hold immense potential for various industrial applications, particularly in the food and pharmaceutical sectors [2,3,4]. Their remarkable biodiversity translates into producing a diverse range of valuable intracellular metabolites. These include proteins, carbohydrates, lipids, and most importantly, carotenoids—a class of compounds renowned for their potent antioxidant properties that significantly contribute to human health [5,6].

Carotenoids are a diverse group of pigments found in all photosynthetic organisms and some non-photosynthetic ones, including microalgae [7]. These vibrant molecules, responsible for the yellow, orange, and red hues in fruits, flowers, and even our skin, are built on a C_40_ isoprenoid backbone. Depending on the specific structure, this backbone can be acyclic or cyclic, with various modifications at its ends [8,9,10]. Common and well-studied carotenoids include lycopene, β-carotene, α-carotene, lutein, zeaxanthin, and β-cryptoxanthin [11,12]. These compounds are not just visually striking; they play an essential role in human health by acting as precursors to vitamin A and providing essential antioxidant protection against cellular damage [13,14]. In recent years, fucoxanthin has garnered significant interest due to its therapeutic potential and ability to address nutritional deficiencies. However, traditional methods for extracting fucoxanthin from brown algae (macroalgae) face limitations. These methods often involve complex and lengthy growth cycles, making large-scale production impractical and driving up costs [15,16].

Microalgae, on the other hand, offer a promising solution for the commercial production of fucoxanthin. These diverse photoautotrophs have the advantage of efficiently accumulating fucoxanthin and achieving high biomass productivity [17]. Unlike macroalgae, their cultivation cycles are significantly shorter and more manageable, paving the way for sustainable and cost-effective production [18]. Among the various fucoxanthin-producing microalgae, *I. galbana*, a member of the Prymnesiophyceae class, takes center stage. Its small size, high digestibility, and rich content of essential nutrients make it a valuable food source for the larvae of shellfish like mussels and clams [19,20,21]. Furthermore, *I. galbana* demonstrates significant potential for successful industrialization due to its ease of cultivation and rapid growth. These advantages have propelled this microalga to the forefront of research and development efforts focused on commercial applications [22,23,24].

Several previous works have explored the optimization of fucoxanthin production from various microalgae, including *Isochrysis galbana*, *Phaeodactylum tricornutum*, and *Tisochrysis lutea* [25,26]. These studies analyzed the impact of different culture media, abiotic parameters (such as temperature, irradiance, and pH), and nutrient sources on fucoxanthin productivity. Other studies have also used sophisticated approaches [27,28], such as response surface methodology and machine learning algorithms, to optimize fucoxanthin production. However, there are still gaps in understanding the complex interactions between the various factors influencing fucoxanthin production and the underlying molecular mechanisms. This research study aims to lay the groundwork for enhancing the cultivation efficiency of *I. galbana* and explore its potential in the pharmaceutical and nutraceutical industries. The primary objective is to optimize fucoxanthin production and the biomass’s overall antioxidant activity. This will be achieved through controlled cultivation experiments examining the influence of different enrichment solutions in the culture media. We will explore the relationships between these factors and their impact on the microalgae.

The first step of our investigation focused on analyzing the correlations between antioxidant activity, fucoxanthin production, carotenoid content, and total phenol levels. Biostatistical tools, such as principal component analysis (PCA) and multiple regression, were used to identify these connections within different culture media formulations designed explicitly for *I. galbana*. Building on these results, the second step consisted of implementing experimental models to optimize fucoxanthin production. The influence of modifiable growth conditions, including choice of growing medium, temperature, pH, lighting intensity, air flow rate, and CO_2_ flow rate, was explored. These parameters were meticulously adjusted to maximize fucoxanthin production while simultaneously assessing their impact on the antioxidant properties of the microalgae. Finally, the last step consisted of an in silico study based on molecular docking to study the molecular interaction between fucoxanthin and proteins with renowned antioxidant potentials in cellular processes.

## 2. Results

### 2.1. Optimization of Growth Parameters

The optimal enriched culture medium for maximizing the biomass production of *I. galbana*, as well as the content of fucoxanthin, carotenoids, total phenols, and antioxidant activity, was selected from several media: 3N-BBM+V medium, ASP-M medium, CHU-10 medium, Conway medium, Erdschreiber medium, Guillard F/2 medium, PM medium, Walne medium, and WC aquatic culture medium. Natural seawater served as a negative control due to its lack of nutrient supplements compared with enriched solutions.

Under standard operating conditions (temperature T = 25 °C, pH = 6.5, light intensity LI = 100 µmol/m^2^/s, air flow AFR = 0.5 L/min, and CO_2_ flow CO_2_FR from 0.1 to 0.2 L/min) over a culture period of 15 days, the results are summarized in Table 1. This table presents data on biomass, antioxidant activity of each extract, and biomolecule contents of each culture medium.

**Figure 1:**
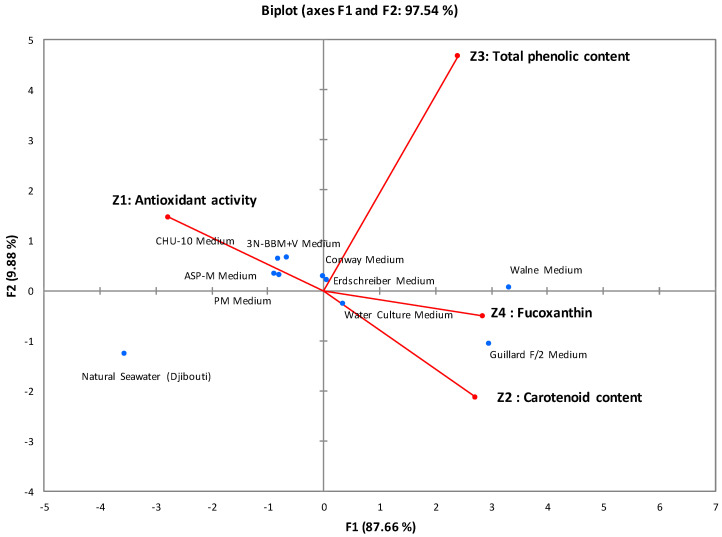
Correlations between antioxidant activity, carotenoid content, total phenolic content, and fucoxanthin content of *I. galbana* cultures. The various circles, represented by distinct colors, symbolize groups sharing common characteristics.

Principal component analysis (PCA) was used to determine the correlations between the studied parameters. Figure 1 graphically illustrates all the correlations obtained, with 97.54% of the variance explained by the first two axes (F1 = 87.66% and F2 = 9.88%). This statistical analysis classified the culture media into three distinct groups:

First group: Comprising Walne medium and Guillard F/2 medium, which showed promising results in terms of biomass, antioxidant activity, and active biomolecule content. The IC50 of antioxidant activity ranged from 285 μg/mL to 304 μg/mL, and the fucoxanthin content reached 7.8 mg/g.

Second group: Including 3N-BBM+V, ASP-M, CHU-10, Conway, PM, WC aquatic culture, and Erdschreiber media, this group showed moderate results in terms of biomass, antioxidant activity, and active biomolecule content. The IC50 of antioxidant activity ranged from 405 μg/mL to 440 ± 2.5 μg/mL, and the fucoxanthin content ranged from 4.6 mg/g to 5.5 mg/g.

Third group: Containing only natural seawater, which served as a negative control with inferior results compared with enriched media. The measured antioxidant activity was 481 μg/mL, and the fucoxanthin content was 2.8 mg/g.

The experimental and statistical results led to the selection of Walne and Guillard F/2 media as the most effective due to their antioxidant power and high fucoxanthin content. These media share essential macronutrients, micronutrients, and vitamins, with Guillard F/2 medium distinguished by including Na_2_EDTA, a chelating agent absent from Walne medium. The differences between these environments are mainly based on the specific proportions of the shared compounds, distinctly influencing the growth and development of microalgae [29,30]. Finally, Walne medium was favored for subsequent studies because of its high fucoxanthin content (7.8 mg/g).

### 2.2. Correlation between Antioxidant Activity and Compound Content

The correlation between antioxidant activity (Z1) and the contents of carotenoids (Z2), total phenolic compounds (Z3), and fucoxanthin (Z4) in cultures of the microalga *I. galbana* was studied using two statistical tools: the matrix of correlation and multiple regression modeling, including multiple linear regression (MLR) and nonlinear multiple regression (MNLR).

The correlation matrix in Table 2 shows the correlation coefficients between each pair of variables. It reveals a strong positive correlation between carotenoid content (Z2) and fucoxanthin (Z4), indicating that fucoxanthin is a significant component of carotenoids. In contrast, antioxidant activity (Z1) showed negative correlations with carotenoid and fucoxanthin content, suggesting that lower values correspond to higher antioxidant activity.

The results of multiple regression modeling (Table S1) show that the two models, MLR and MNLR, perform very well with high coefficients of determination (R^2^) of 0.954 and 0.986, respectively. However, the nonlinear model (MNLR) shows greater explanatory capacity, indicating a more complex relationship between variables, which is better captured by this model. In terms of predictive accuracy, MNLR has a mean square error (MCE) of 156.573, lower than that of MLR (262.950), confirming better accuracy of the nonlinear model. Similarly, the root mean square error (RMCE) is lower for MNLR (12.513) than for MLR (16.216), further emphasizing the superior fit of the nonlinear model to the observed data.

Figure S1 illustrates the linearity of the predictive results compared with the experimental results, demonstrating that MNLR aligns better with the experimental data than MLR. In conclusion, the results indicate that the relationships between antioxidant activity (Z1) and the contents of carotenoids (Z2), total phenolic compounds (Z3), and fucoxanthin (Z4) are well correlated, and nonlinear models are more appropriate for capturing the complexity of these relationships in *I. galbana* cultures.

### 2.3. Optimization of Experimental Conditions by Experimental Design

The experiments were planned to optimize the experimental conditions aimed at maximizing the production of fucoxanthin following the statistical tool of the experimental plan, taking into consideration five operational parameters: temperature, pH, light intensity, air flow rate, and CO_2_ flow rate. Table S2 presents the results obtained for the fucoxanthin content for each of the 32 tests according to the polynomial model of full factorial designs at two levels (−1 and +1).

A first-order polynomial mathematical model was developed from the results obtained, including the main factors and higher-order interactions up to the fourth order between these five parameters. The coefficients of the fucoxanthin production model equation are listed in Table S3. The average yield achieved by the optimization of conditions was 7.93 mg/g with a coefficient of variation of 1.11%.

The statistical model derived from the experimental design is extremely accurate and well fitted to the experimental data. The statistical parameters presented in Table S4 (coefficient of determination: R^2^ = 0.99, adjusted R^2^: R^2^_adj_ = 0.99, predicted R^2^: R^2^_pre_ = 0.91, and adequate precision: P = 87.36%) indicate that the model explains a large part of the observed variation and has good predictive ability for new observations.

Table S5 presents the analysis of variance (ANOVA), which is used to assess the significance of the effects of different factors in the experimental design. The model is considered significant with an F value of 386.15, with only a 4.02% probability that such a high F value is due to chance. *p*-Values of less than 0.05 indicate that some model terms are substantial—D, E, AE, CE, DE, ACE, CDE, and BCDE—while others are not.

Finally, Figure S2 graphically illustrates the main interactions, which are significant contributors to the variation in fucoxanthin concentration, by showing interactions in one dimension (D and E), two dimensions (AE, CE, and DE), and three dimensions (ACE and CDE).

### 2.4. In Silico Study

**Figure 2:**
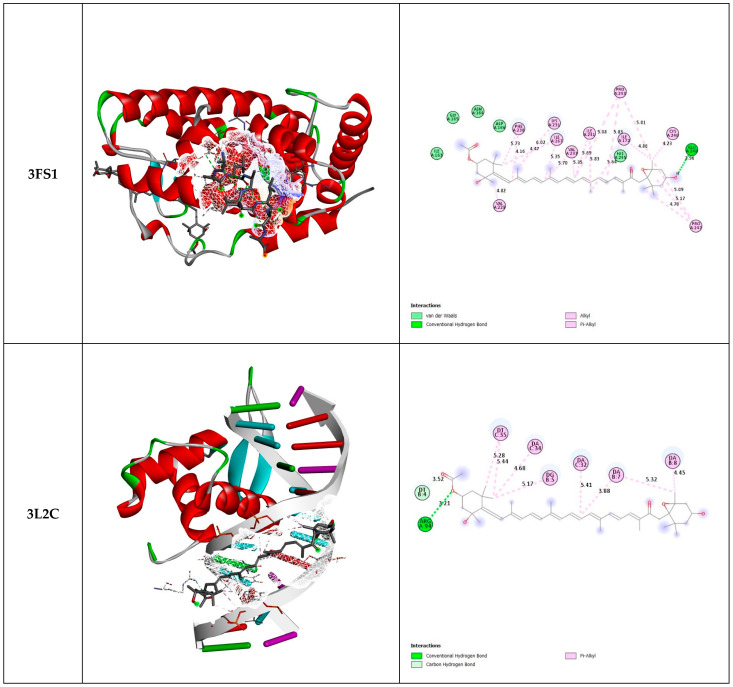

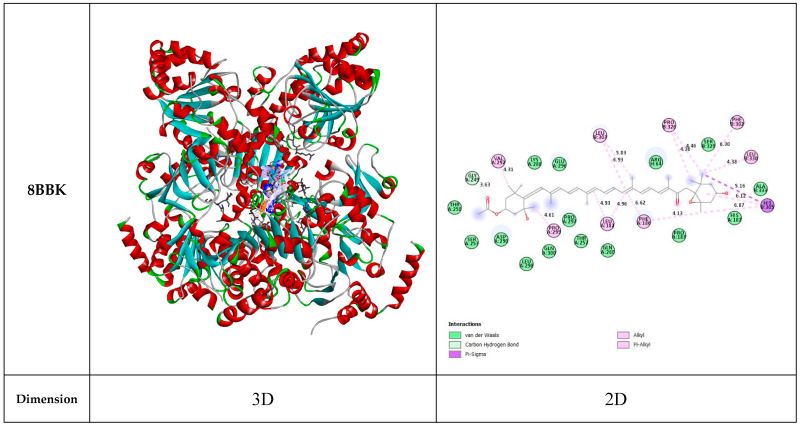
Three-dimensional and two-dimensional docked views of fucoxanthin with 3FS1, 3L2C, and 8BBK proteins, respectively.

The results of molecular docking of fucoxanthin with the three selected proteins (3FS1, 3L2C, and 8BBK) are shown in Figure 2 and Table 3.

The three proteins studied play an important role in antioxidant processes, as follows:

The 3FS1 protein, corresponding to HNF4a (hepatocyte nuclear factor 4 alpha), plays an essential role in the cellular antioxidant process by modulating the expression of genes associated with detoxification and the response to oxidative stress. As a transcription factor, HNF4a induces the expression of genes encoding antioxidant enzymes such as superoxide dismutase (SOD), catalase, and glutathione-S-transferases. These enzymes neutralize reactive oxygen species (ROS) and attenuate oxidative damage in liver cells and other tissues where HNF4a is expressed [31,32].

The 3L2C protein, identified as FOXO4 (forkhead box O4), plays a role in the antioxidant response by regulating the expression of genes encoding antioxidant enzymes and DNA repair proteins. In response to various cellular signals, including oxidative stress, FOXO4 migrates to the cell nucleus, where it binds to specific DNA sequences and activates the transcription of genes such as SOD, glutathione peroxidase, and other enzymes and antioxidants. This action helps neutralize ROS and restore redox balance in cells, thus reducing oxidative damage [33,34,35].

As for the 8BBK protein, corresponding to Sirt3 (Sirtuin 3), Sirt3 participates in the antioxidant process by deacetylating and activating key enzymes involved in defense against oxidative stress. Notably, Sirt3 deacetylates and activates SOD2 (MnSOD) localized in mitochondria, which increases the activity of this critical antioxidant enzyme. SOD2 converts superoxide to hydrogen peroxide, which is then broken down into water and oxygen by other enzymes. In addition, Sirt3 regulates other proteins involved in the mitochondrial antioxidant response, thereby preserving the functional integrity of mitochondria and reducing cellular oxidative stress [36,37].

Fucoxanthin exhibits a stronger interaction with 3L2C and 8BBK proteins compared with 3FS1, indicating a higher affinity of fucoxanthin for FOXO4 (3L2C) and Sirt3 (8BBK) compared with HNF4a (3FS1). This higher affinity suggests that fucoxanthin has an increased propensity to bind FOXO4 and Sirt3. Additionally, higher ligand efficiency implies that the ligand performs better per unit size in binding to the protein. In this context, fucoxanthin demonstrates slightly higher efficacy when interacting with FOXO4 (3L2C) and Sirt3 (8BBK) compared with HNF4a (3FS1).

**Figure 3:**
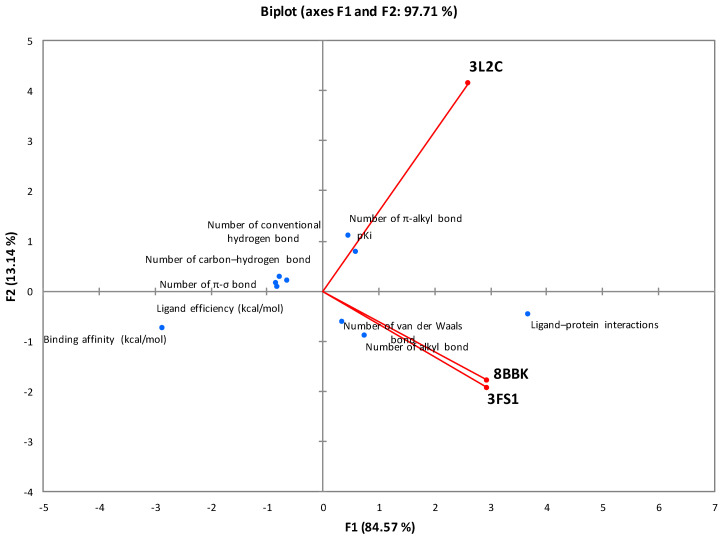
Correlation between the molecular docking parameters between fucoxanthin and the proteins studied.

The correlation with the principal component analysis (PCA) of all thermodynamic parameters and interaction bonds of the different types of molecular docking is presented in Figure 3. The analysis of this representation, based on the F1 and F2 axes (97.71%), proves that the specific interactions between fucoxanthin and the HNF4a (3FS1) and Sirt3 (8BBK) proteins are well correlated. On the other hand, the interactions of these last two proteins are not correlated with FOXO4, which suggests that fucoxanthin adopts two different mechanisms depending on the proteins studied.

## 3. Discussion

Fucoxanthin is widely recognized for its bioactive potential and substantial antioxidant activity, making it a compound with many industrial applications. These advantages are enhanced by continued advances in extraction and quantification techniques and a better understanding of its biological properties [38].

The quantification of fucoxanthin, an abundant carotenoid in microalgal biomass, particularly in *I. galbana*, is essential for its industrial and therapeutic applications [39]. In the pharmaceutical sector, it is used to develop food supplements and drugs due to its beneficial antioxidant, anticancer, anti-diabetes, anti-inflammatory effects, etc. [40,41,42,43]. Nutraceuticals incorporate it into functional food products for its health properties. In the cosmetic field, it is incorporated into skin care formulations for its anti-ageing effects and ability to protect against UV damage. In agriculture, its bioactive properties make it a potential candidate as a biofertilizer and plant protective agent [44].

The main objective of this study was to quantify fucoxanthin and valorize its antioxidant properties as an abundant carotenoid in *I. galbana* while maximizing its extraction and use in various industrial sectors. This included optimizing production and standardizing and establishing accurate quantification methods to ensure the quality and consistency of fucoxanthin products, facilitating their introduction to the market. Furthermore, the exploitation of the antioxidant properties of fucoxanthin has been explored for the development of new products while promoting the use of natural and renewable sources to support sustainable industrial practices.

The significant results of this study showed that Walne and Guillard F/2 culture media were the most effective in producing fucoxanthin in *I. galbana* culture due to their high antioxidant potential and increased fucoxanthin content. Correlation analysis revealed a strong association between antioxidant activity and fucoxanthin and carotenoid content and a moderate correlation with total phenols. Regression models, whether linear or nonlinear, have been used to model these relationships, with the nonlinear model showing a better ability to explain and predict antioxidant activity, suggesting a more accurate consideration of complex interactions. Using experimental designs, optimal cultivation conditions were identified, including temperature, pH, light intensity, air flow rate, and CO_2_ flow rate, with particular attention paid to the last two parameters to influence the production of fucoxanthin significantly. The developed mathematical models demonstrated robust predictive ability and offer promising prospects for future optimization of fucoxanthin production in a sustainable industrial setting.

Recent works (Table 4) that have received good recognition in the literature concerning optimizing fucoxanthin production are numerous, among which the most important over the last 5 years are presented below.

In our study on fucoxanthin production from *I. galbana*, we explored various culture media and advanced analytical approaches to optimize the production of this bioactive pigment. Compared with previous studies, our analysis reveals several important and divergent aspects.

Medina et al. (2019) [45] demonstrated that methanol and ethanol are the most effective solvents to extract fucoxanthin from *I. galbana*, with significantly higher yields than petroleum ether and n-hexane. Their optimization of extraction time also highlighted the potential of *I. galbana* as a natural source of fucoxanthin. In contrast, our study shows that optimization of culture conditions and media, such as Walne and Guillard media, also plays a critical role in enhancing fucoxanthin production.

Gao et al. (2020) [46] optimized fucoxanthin production in *Tisochrysis lutea* by adjusting culture and irradiance parameters. They observed that higher dilution rates and specific irradiance maximized production. Our results support these observations to some extent, but our multidimensional approach allowed for further optimization by combining culture, statistical analysis, and bioinformatics validation techniques, which could provide additional insights for large-scale production.

Pereira et al. (2021) [47] illustrated seasonal variations in fucoxanthin production, showing that *Phaeodactylum tricornutum* performs better in autumn/winter, while *T. lutea* performs better in spring/summer. While their study highlights the feasibility of continuous production, our study highlights the impact of specific growth conditions and analytical approaches on maximizing production.

The works of Mohamadnia et al. (2021) [48] and Mohamadnia et al. (2022) [49] on optimizing fucoxanthin production using response surface methodology confirm the importance of precise culture conditions. Their approach identified optimal parameters for *T. lutea*, which is consistent with our observation that growth conditions play an essential role. However, our use of in silico analyses to study the molecular interactions of fucoxanthin added an additional dimension to understanding its bioactive properties.

McElroy et al. (2023) [50] implemented an integrated biorefinery approach to extract fucoxanthin and other compounds from *Saccharina latissima*. Their process integration to reduce the ecological footprint is particularly relevant, although our study mainly focused on optimizing culture conditions and validating the results using bioinformatics approaches.

A study conducted by Xia et al. (2023) [51] showed that intermittent CO_2_ supply promotes better fucoxanthin accumulation in *I. galbana*, a result in agreement with our observation that optimization of culture conditions is crucial for increased production. Furthermore, a study conducted by Bo et al. (2023) [52] revealed that spermidine positively influences fucoxanthin biosynthesis, highlighting the importance of biological factors in the production of this pigment.

Finally, Garcia-García et al. (2024) [53] explored the use of green solvents for fucoxanthin extraction, highlighting the advantages of modern techniques over traditional methods. This reinforces our conclusion that the use of advanced methods and solvents is essential to optimize extraction while preserving the bioactivity of the extracts.

In conclusion of this discussion, our study, through its multidimensional approach integrating the optimization of growth conditions, advanced statistical analysis, and bioinformatics validation, provides a more comprehensive and innovative overview for fucoxanthin production. These results contribute to a better understanding of the parameters influencing the production of this pigment and offer promising prospects for its industrial application.

**Figure 4:**
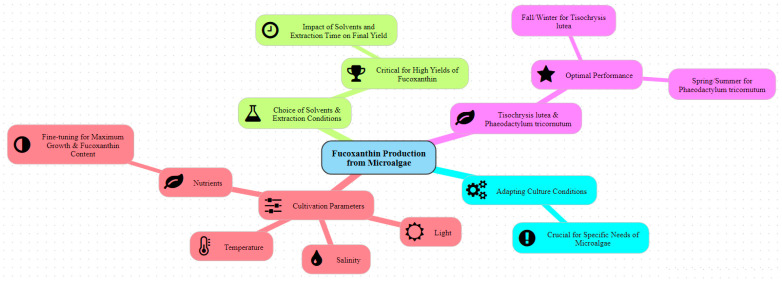
The main factors in the production of fucoxanthin from microalgae (created with www.map-this.com, accessed on 3 June 2024).

The mentioned research on the production of fucoxanthin from microalgae has focused its attention on three key aspects: optimization of environmental variations; precision in the adjustment of culture parameters such as temperature, lighting, pH, and salinity and the sources of nutrients, O_2_ and CO_2_; and the improvement of extraction processes using specific solvents and optimized conditions (Figure 4) [54,55,56,57,58]. These studies highlight the remarkable importance of adapting culture conditions to maximize productivity and fucoxanthin concentration in microalgae cultures, considering seasonal variations, while judiciously choosing extraction methods to ensure high yields of this bioactive compound.

Molecular docking results of fucoxanthin with 3FS1 (HNF4a), 3L2C (FOXO4), and 8BBK (Sirt3) proteins revealed distinct interactions, highlighting a marked preference of fucoxanthin for FOXO4 and Sirt3 over HNF4a. These proteins have remarkable biochemical processes in the fine regulation of antioxidant responses and defense against oxidative stress, as evidenced by their ability to influence the expression of enzymes such as superoxide dismutase and glutathione peroxidase [59,60,61]. The analysis highlighted the specificity of these interactions, revealing that fucoxanthin adopts diverse mechanisms depending on the target proteins. This functional diversity reinforces the therapeutic potential of fucoxanthin as an antioxidant. By optimizing the production of fucoxanthin from *I. galbana* under specified conditions, this study laid a solid foundation for future biotechnological applications aimed at exploiting the promising antioxidant properties of this natural compound.

## 4. Material and Methods

### 4.1. Cultivation of the Microalgae I. galbana

The marine microalgae strain *I. galbana* was isolated from the coast of Tadjoura in Djibouti (1°46′58.084″ N, 42°53′1.667″ E). Initial isolation was conducted using Falcon conical tubes and cell isolation through micropipettes under a microscope after successive dilutions. The microalgae were then cultured in a liquid medium and on agar plates. Cultivation was carried out in seawater collected from the same location, which was sterilized and enriched with various culture media, including 3N-BBM+V medium, ASP-M medium, CHU-10 medium, Conway medium, Erdschreiber medium, Guillard F/2 medium, PM medium, Walne medium, and WC aquatic culture medium. Some of these media were supplemented with silicate (sodium metasilicate, Sigma-Aldrich 307815, Missouri, United States), particularly CHU-10 medium, Conway medium, and PM medium.

Initially, *I. galbana* was cultured at 25 °C in shakers using natural seawater under continuous illumination of 150 μmol m^−2^ s^−1^. Growth was monitored by measuring the optical density at 680 nm (Shimadzu UV-1601 spectrophotometer, Kyoto, Japan) every 2 days over a growth period of approximately 10 days. In the subsequent phase, cultures were sub-cultured according to the specific experimental conditions (selected medium, temperature, pH, lighting, flow rate, and CO_2_ flow rate) until the biomass reached the stationary phase (15th day of culture). Biomass was then harvested by centrifugation at 2000 rpm (Thermo Scientific™ Megafuge™ 8, Waltham, MA, USA) for 10 min, freeze-dried (Christ Alpha-2–4 LDPlus, Osterode am Harz, Germany), and stored at −20 °C.

### 4.2. Procedure for Obtaining Extracts for Analyses

The extraction process was performed using the maceration method. For this, 1 g of *I. galbana* biomass was extracted with 100 mL of a methanol/chloroform mixture (1:1, *v*/*v*) for 12 h at room temperature in complete darkness. This extraction was repeated three times, and all extracts were combined. The pooled extracts were then filtered through Whatman No. 4 filter paper and concentrated under reduced pressure using a rotary evaporator. The final extracts were stored in amber glass vials at −20 °C until further use [62].

### 4.3. Determination of Antioxidant Activity by the DPPH Method 

The antioxidant activity by scavenging DPPH free radicals was evaluated following the method of Flieger et al. (2020) [63]. A stock solution of each extract was prepared in methanol at a concentration of 20 mg/mL. Then, 20 µL of each extract was added to 180 µL of DPPH radical solution (60 µM) in 96-well plates, resulting in final concentrations of 50, 100, 200, 500, and 1000 µg/mL. The samples were shaken to ensure thorough mixing. Since some colored extracts can absorb at 520 nm, a control (blank sample) was prepared by adding 20 μL of each sample solution to 180 μL of methanol. The absorbance of the samples was measured at 30, 60, and 120 min at 520 nm using methanol as a blank in a Shimadzu UV-1601 spectrophotometer. The IC_50_ (50% inhibitory concentration) was calculated to compare the free radical scavenging efficiency. DPPH radical scavenging activity was calculated using the following equation:

$$Scavenging\;effect\left(\%\right)=100\times \left[1-\left(\frac{AS-AB}{AC}\right)\right]$$

*AS*: Sample absorbance is the absorbance of the methanolic solution of DPPH in the presence of all the extracts and the standard.

*AB*: Blank absorbance is the absorbance of the sample of extracts in methanol (without DPPH to subtract the absorbance of the colored extracts).

*AC*: Control absorbance is the absorbance of the methanolic solution of DPPH.

### 4.4. Estimation of Carotenoid Content

A method described by Zhou et al. (2020) [64] was employed to determine the carotenoid content of *I. galbana* samples. Approximately 2 g of each sample was mixed with 25 mL of methanol, vortexed for 10 min, and filtered through Whatman No. 1 filter paper. The filtrate was fractionated with 20 mL of petroleum ether and subsequently washed with 100 mL of distilled water. Any residual water was removed using Whatman No. 1 filter paper coated with 5 g of anhydrous sodium sulfate. The extract volume was adjusted to 25 mL with ethanol, and the absorbance was measured at 450 nm using a spectrophotometer.

### 4.5. Total Phenolic Content

Total phenolic content was determined using the Folin–Ciocalteu reagent, according to the procedure described by Pauliuc (2020) [65]. Each sample (1 mg/mL) was mixed with 5 mL of reagent, diluted (1:10 *v*/*v*) with water, and mixed with 4 mL of 7.5% sodium carbonate. The total phenolic content was measured at 750 nm using a spectrophotometer. Gallic acid was used as a standard (0 to 200 mg/mL). The result was expressed as mg of gallic acid equivalent (GAE) per 100 g of sample.

### 4.6. Quantification of Fucoxanthin

Fucoxanthin quantification was carried out according to the method described by Li et al. (2021) [66] using high performance liquid chromatography (HPLC) coupled with UV detection. The analyses were carried out on an Agilent 1200 HPLC System (Agilent Technologies), including a quaternary pump and a diode array detector (DAD). Chromatographic separation was carried out using a YMC-Pack ODS-A, C18 column (250 mm × 4.6 mm, 5 µm). The column temperature was regulated at 30 °C to optimize the separation of the analytes. The mobile phase, consisting of a methanol–water mixture (80:20, *v*/*v*), was supplied at a constant flow rate of 1 mL/min. Samples were injected at a volume of 20 µL. The detection of fucoxanthin was performed at a wavelength of 450 nm, specifically chosen for this analysis. Standard solutions of fucoxanthin were prepared by dissolving the compound in methanol to obtain a stock solution, which was then diluted to prepare standard solutions with concentrations ranging from 0.5 to 12 ppb; hence, Figure S3 presents the calibration curve made for this quantification. The microalgae extracts were also diluted in methanol and injected to a volume of 20 μL under the same operating conditions to quantify fucoxanthin. Before being injected into the HPLC system, all solutions were degassed and filtered through a 0.22 µm membrane to remove particles that could damage the column or disrupt the analysis.

### 4.7. Statistical Studies

Numerical data were collected from three replicates for each test to ensure the accuracy of the results. Type A uncertainty assessment was performed for the statistical analysis of numerical data. The tests were then subjected to Student’s *t*-test (*p* < 0.05) to assess their significance. To detect significant differences between groups of samples, analysis of variance (ANOVA) was performed, followed by Tukey’s test for multiple comparisons. This test was used to identify significant variations between groups and provide a detailed analysis of the observed differences.

For correlation studies, data modeling was conducted using XLSTAT version 2016, employing several statistical correlation methods to determine relationships between parameters. Principal component analysis (PCA) was utilized to transform a set of correlated variables into a reduced number of uncorrelated principal components [67]. This analysis evaluated the correlation between the enrichment of culture media of the microalga *I. galbana* and antioxidant activity, as well as the contents of carotenoids, total phenols, and fucoxanthin, classifying the environments according to these studied parameters. Additionally, the correlation between antioxidant activity, carotenoids, total phenols, and fucoxanthin was analyzed using simple linear regressions between each pair of parameters and multiple linear regression (MLR) and multiple nonlinear regression (MNLR) [68]. These statistical approaches aimed to establish the relationships between antioxidant activity and the contents of carotenoids, total phenols, and fucoxanthin. Design-Expert 13 was used for all calculations and graphical representations for optimization studies utilizing experimental designs. The methodology aimed to minimize the experimental conditions for producing fucoxanthin from the microalga *I. galbana*.

The experimental design used was a full factorial design based on the following five X_i_ factors:

Factor 1 = T: temperature (25 °C and 30 °C);
Factor 2 = pH: pH (6.5 and 7.5);
Factor 3 = light intensity: LI (100 µmol/m^2^/s and 500 µmol/m^2^/s);
Factor 4 = air flow rate: AFR (0.5 L/min and 1.0 L/min);
Factor 5 = CO_2_ flow rate: CO_2_FR (0.1–0.2 L/min).

Each factor was studied at two levels—high (+1) and low (−1)—resulting in 2^5^ = 32 trials in total. The polynomial model included the following:

$$Fucoxanthin\;\left(mg/g\right)=a_{0}+\sum_{i=1}^{n}a_{i}X_{i}+\sum_{i=1}^{n}\sum_{j=1}^{n-1}a_{ij}X_{i}X_{j}+\sum_{i=1}^{n}\sum_{j=1}^{n-1}\sum_{k=1}^{n-2}a_{ijk}X_{i}X_{j}X_{k}+\sum_{i=1}^{n}\sum_{j=1}^{n-1}\sum_{k=1}^{n-2}\sum_{l=1}^{n-3}a_{ijkl}X_{i}X_{j}X_{k}X_{L}+a_{ijklm}X_{i}X_{j}X_{k}X_{l}X_{m}$$

A mean: *a*_0_;
5 main effects for each factor: *a_i_*;
10 interactions of order 2: *a_ij_*;
10 interactions of order 3: *a_ijk_*;
5 interactions of order 4: *a_ijkl_*;
1 interaction of order 5: *a_ijklm_*.

All values obtained are presented as mean ± uncertainty, with a significance level of 5% for each experiment, as determined by statistical analysis using the Student’s *t*-test. Each experimental variant was tested in triplicate.

### 4.8. In Silico Study 

This in silico study used molecular docking methodology to perform a virtual screening of the crystal structures of a few antioxidant proteins available in the RCSB database, including PDB IDs (3FS1, 3L2C, and 8BBK). The choice of these crystal structures for molecular docking was guided by their relevance to the study objectives aimed at evaluating the antioxidant activity of fucoxanthin. These proteins were selected based on their potential involvement in antioxidant processes, as confirmed by previous works, as well as their structural availability in the RCSB database, ensuring their validity and feasibility for comparative computational analysis [69,70,71]. This approach allowed the investigation of specific interactions with proteins relevant to this study, thereby contributing to a cross-sectional understanding of the potential effects of fucoxanthin. To carry out this analysis, several software tools were employed, including MGLtools 1.4.6, Autodock 4.0, Autogrid 4.0, BIOVIA Discovery Studio Visualizer 2.5, ChemBiodraw Ultra 12.0, and Chemdraw 3D 10.0. Initially, protein structures were prepared using the BIOVIA Discovery Studio Visualizer to remove heteroatoms, co-crystallized ligands, and solvents to optimize conditions for docking. Autodock tools were then used to assign appropriate polar charges and generate optimized pdbqt files for each protein structure. For fucoxanthin, the structure was drawn with ChemDraw Ultra, then energetically minimized with Chem 3D Pro before being converted to pdbqt format via OpenBabel GUI. Structure-based virtual screening was performed with Autodock 4, docking fucoxanthin independently into the active site of each target protein. Ligand–protein interactions were visualized and analyzed with the BIOVIA Discovery Studio Visualizer. To validate the results, root mean square deviation (RMSD) values were calculated, ensuring that accepted poses had RMSD values of less than 2.0 for ligands redocked by co-crystallization.

## 5. Conclusions

This study successfully optimized the production of fucoxanthin from cultures of *I. galbana*. F/2 media were identified as the most effective, promoting high fucoxanthin content and improving the overall antioxidant potential of the alga. Analyses demonstrated close correlations between fucoxanthin and total carotenoids, linked to antioxidant activity. A specific statistical model (MNLR) effectively captured the complex interactions between various factors influencing fucoxanthin production. Furthermore, the experiments identified air flow rate and CO_2_ flow rate as crucial factors for maximizing fucoxanthin yields. Computational molecular docking analyses showed that fucoxanthin binds most efficiently to FOXO4 and Sirt3, two proteins playing critical roles in the antioxidant response and regulation of key enzymes against oxidative stress. Furthermore, the results confirmed these specific interactions, highlighting the propensity of fucoxanthin to adopt different mechanisms depending on the targeted proteins. These findings suggest the significant potential of fucoxanthin in antioxidant and therapeutic applications. In conclusion, all the data from this work validate the robustness of the developed model, which is very promising for the future optimization of fucoxanthin production in biotechnological applications.

## Figures and Tables

**Table 1:** Dry biomass, antioxidant activity, carotenoid content, total phenolic content, and fucoxanthin content of cultures of the microalga *I. galbana*.

Culture Medium	Dry Weight Biomass (g/L)	DPPH IC_50_ (μg/mL)	Carotenoids Content (mg/g)	Total Phenolic Content (mg/100 g)	Fucoxanthin (mg/g)
Natural Seawater (Djibouti)	0.65 ± 0.12 ^a^	481 ± 25 ^a^	9.9 ± 0.2 ^a^	12.0 ± 1.1 ^a^	2.8 ± 0.5 ^a^
3N-BBM+V Medium	0.88 ± 0.17 ^a,b,c^	438 ± 20 ^a,b^	11.5 ± 1.3 ^b^	20.0 ± 1.8 ^b^	4.6 ± 0.8 ^b^
ASP-M Medium	0.82 ± 0.16 ^a,b,c^	435 ± 25 ^a,b^	11.7 ± 1.2 ^b^	19.2 ± 1.7 ^b^	4.8 ± 0.7 ^b^
CHU-10 Medium	0.91 ± 0.18 ^b,c^	440 ± 25 ^a,b^	11.5 ± 1.1 ^b^	20.1 ± 1.8 ^b^	5.0 ± 0.8 ^b^
Conway Medium	0.92 ± 0.18 ^b,c^	415 ± 25 ^b,c^	13.1 ± 1.5 ^b,c^	20.3 ± 2.0 ^b^	5.0 ± 0.6 ^b^
Erdschreiber Medium	0.95 ± 0.19 ^b,c^	405 ± 20 ^c^	12.8 ± 1.8 ^b,c^	20.1 ± 2.1 ^b^	5.2 ± 0.5 ^b^
Guillard F/2 Medium	1.22 ± 0.21 ^c,d^	304 ± 15 ^d^	16.6 ± 2.4 ^d^	20.5 ± 1.8 ^b^	7.8 ± 1.0 ^c^
PM Medium	0.82 ± 0.16 ^a,b,c^	421 ± 30 ^b,c^	11.2 ± 1.2 ^a,b^	19.1 ± 1.8 ^b^	4.6 ± 0.5 ^b^
Walne Medium	1.28 ± 0.22 ^c,d^	285 ± 15 ^e^	15.4 ± 1.8 ^d^	23.6 ± 2.5 ^b,c^	7.8 ± 0.8 ^c^
Water Culture Medium	1.15 ± 0.20 ^c,d^	413 ± 25 ^b,c^	14.2 ± 1.6 ^c,d^	19.4 ± 1.8 ^b^	5.5 ± 0.5 ^b^

Different letters in the same row indicate significant differences according to Tukey’s test (*p* < 0.05).

**Table 2:** Correlation matrix.

Matrix	Z2	Z3	Z4	Z1
Z2	1			
Z3	0.660	1		
Z4	0.943	0.794	1	
Z1	−0.913	−0.706	−0.970	1

Z1: antioxidant activity; Z2: carotenoid content; Z3: total phenolic content; and Z4: fucoxanthin.

**Table 3:** Qualitative and energetic characteristics of molecular docking of fucoxanthin with antioxidant activity proteins.

Proteins	3FS1	3L2C	8BBK
Binding affinity (kcal/mol)	−7.3	−9.4	−9.3
p*Ki*	5.35	6.89	6.82
Ligand efficiency (kcal/mol)	0.1521	0.1958	0.1938
Ligand–protein interactions	25	10	31
Number of π-σ bond	0	0	1
Number of alkyl bond	15	0	10
Number of π-alkyl bond	4	8	4
Number of conventional hydrogen bond	1	1	1
Number of carbon–hydrogen bond	0	1	0
Number of van der Waals bond	5	0	15

**Table 4:** Main works of optimization of fucoxanthin production.

Work	Reference	Year	Study Objective	Methodology	Key Results	Quantity of Fucoxanthin	Implications
1	Médine et al. [45]	2019	Examine the extraction of fucoxanthin from *I. galbana* for obesity prevention.	Comparison of solvents (methanol, ethanol, petroleum ether, n-hexane). Optimization of extraction time.	Best yields with methanol (6.282 mg/g DW) and ethanol (4.187 mg/g DW). Optimal extraction in 10 min with 100% ethanol.	6.282 mg/g DW (methanol), 4.187 mg/g DW (ethanol)	*I. galbana* is a promising source of fucoxanthin for the food industry.
2	Pereira et al. [46]	2021	Optimize industrial-scale fucoxanthin production.	Cultivation in 15 m^3^ tubular photobioreactors. Seasonal comparison between *P. tricornutum* and *T. lutea*.	*P. tricornutum* achieved 2.87 g DW L^−1^ and 0.7% DW fucoxanthin (7 mg/g DW) in fall/winter. *T. lutea* was more productive in spring/summer.	7 mg/g DW	Feasibility of continuous fucoxanthin production year-round.
3	Gao et al. [47]	2020	Optimize fucoxanthin production in *T. lutea.*	Batch and continuous experiments, adjusting parameters like temperature, irradiation, and dilution rate.	Maximum productivity at 30 °C and 300 μmol m^−2^ s^−1^. High dilution rates (0.53 and 0.80 d^−1^) and light absorption of 2.23 mol m^−2^ d^−1^ favored high fucoxanthin content.	16.39 mg/g DW	Light absorption can predict fucoxanthin content.
4	Mohamadnia et al. [48]	2021	Optimize fucoxanthin production in *T. lutea* using response surface methodology.	Adjustment of culture conditions in 1 L batch photobioreactors with polynomial second-order modeling.	Optimal conditions: salinity 36.27 g L^−1^, starch 3.90 g L^−1^, nitrate 0.162 g L^−1^.	79.4 mg/g DW	Optimization of culture parameters to maximize fucoxanthin production.
5	Mohamadnia et al. [49]	2022	Refine fucoxanthin production using response surface methodology (RSM).	Adjustment of concentrations of glutamic acid, trisodium citrate, succinic acid, sodium aspartate, and pyruvate.	Optimal concentrations: sodium aspartate 7.5 mM, sodium pyruvate 7.5 mM, glutamic acid 3.29 mM. Productivity of 22.4 mg L^−1^ day^−1^.	22.4 mg L^−1^ day^−1^	Metabolic optimization strategies to increase fucoxanthin production.
6	McElroy et al. [50]	2023	Integrate biorefining to valorize *Saccharina latissima* biomass.	Optimized extraction of fucoxanthin at 40 MPa. Integration with mannitol and alginate extraction. Life cycle analysis.	4.15% yield for fucoxanthin. Extraction of 67.27% to 69.38% of alginates. Reduction in environmental impact identified.	41.5 mg/g DW	Integrated biorefining processes to reduce environmental footprint.
7	Xia et al. [51]	2023	Assess the impact of CO_2_ concentration and frequency on fucoxanthin production.	Comparison of continuous and intermittent CO_2_ supply at different concentrations.	Continuous CO_2_ at 5% achieved maximum biomass productivity (0.33 g L^−1^ day^−1^). Intermittent CO_2_ at 5% optimized fucoxanthin accumulation.	0.56 mg/g DW	Improved fucoxanthin accumulation with intermittent CO_2_ supply.
8	Bo et al. [52]	2023	Study the effect of spermidine on fucoxanthin biosynthesis in *Isochrysis* sp.	Addition of spermidine under different light intensities and assessment of cell proliferation and pigment synthesis.	Optimal cell density of 5.40 × 10^6^ cells/mL after 11 days. Maximum diadinoxanthin (1.09 mg/g DW) and fucoxanthin under low light intensity.	6.11 mg/g DW	Spermidine enhances fucoxanthin production and mitigates photosystem damage under high light intensity.
9	Garcia-García et al. [53]	2024	Explore extraction of fucoxanthin and DHA from *T. lutea* using green solvents.	Use of green solvents and advanced extraction techniques, such as ultrasonic-assisted extraction with 2-methyltetrahydrofuran and ethanol.	High extraction yields of fucoxanthin and DHA. 2-Methyl-tetrahydrofuran-enriched extracts showed better composition.	High (exact quantity not provided)	Advanced extraction techniques to preserve bioactivity of extracts.
10	Manochkumar et al. [54]	2024	Optimize fucoxanthin production using machine learning.	Development of a machine learning model to predict fucoxanthin yield based on phytohormone supplementation.	Random Forest and ANN models showed improved accuracy with hormone descriptors.	-	Combining UV spectrometry and ML algorithms for precise fucoxanthin predictions and production optimization.
11	This work	-	Optimize fucoxanthin production from *I. galbana* and validate antioxidant potential.	Test various culture media. Evaluate antioxidant potential. Use PCA and regression models. Optimize growth conditions (air flow rate and CO_2_ flow). Perform molecular docking analysis.	Walne and Guillard media most effective. Strong correlation between antioxidant activity and fucoxanthin. Air flow rate and CO_2_ flow are key factors. Fucoxanthin interacts with antioxidant proteins.	13.4 mg/g DW	Improved production methods. Validated antioxidant benefits. Insights for future research and applications.

## Data Availability

Data are contained within the article.

## Supplementary Materials

The following supporting information can be downloaded at: https://www.mdpi.com/article/10.3390/md22080358/s1, Figure S1. displays a validation graph depicting the predicted antioxidant activity of the marine microalga *I. galbana*; Figure S2. Illustrates the representations of fucoxanthin across one-dimensional, two-dimensional, and three-dimensional formats; Figure S3. Calibration curve of fucoxanthin by HPLC-UV; Table S1. Statistical parameters of the multiple regression of antioxidant activity as a function of the content of carotenoids, total phenolic compounds and fucoxanthin in cultures of the microalga *I. galbana*; Table S2. Fucoxanthin is used for each test in all experiments according to the design of the experiments; Table S3. Coefficients of optimized models for fucoxanthin production; Table S4. Statistical studies of the model; Table S5. ANOVA for the selected factorial model.
