# Supplementary material for: Production of Fucoxanthin from Microalgae Isochrysis galbana of Djibouti: Optimization, Correlation with Antioxidant Potential, and Bioinformatics Approaches
Source: Mar Drugs. 2024 Aug 6;22(8):358. doi: 10.3390/md22080358 (PMC11355383; doi:10.3390/md22080358)
Supplement: Supplementary file 1 [file marinedrugs-22-00358-s001.zip › marinedrugs-3122343-supplementary.pdf]

Supplement information

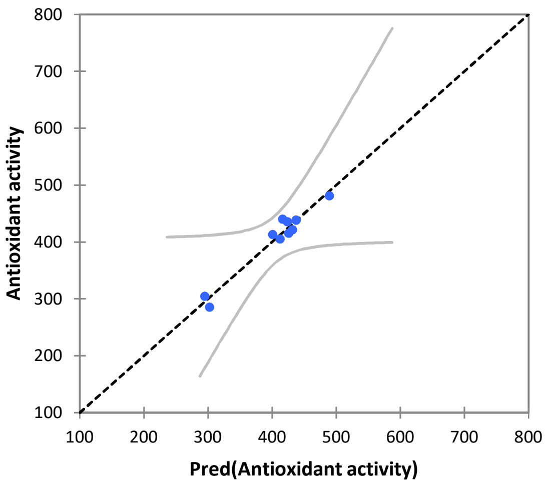

MLR

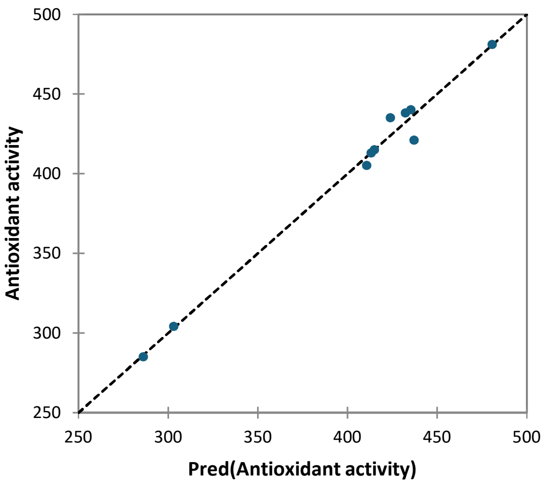

MNLR

Figure S1. displays a validation graph depicting the predicted antioxidant activity of the marine microalga *I. galbana.*

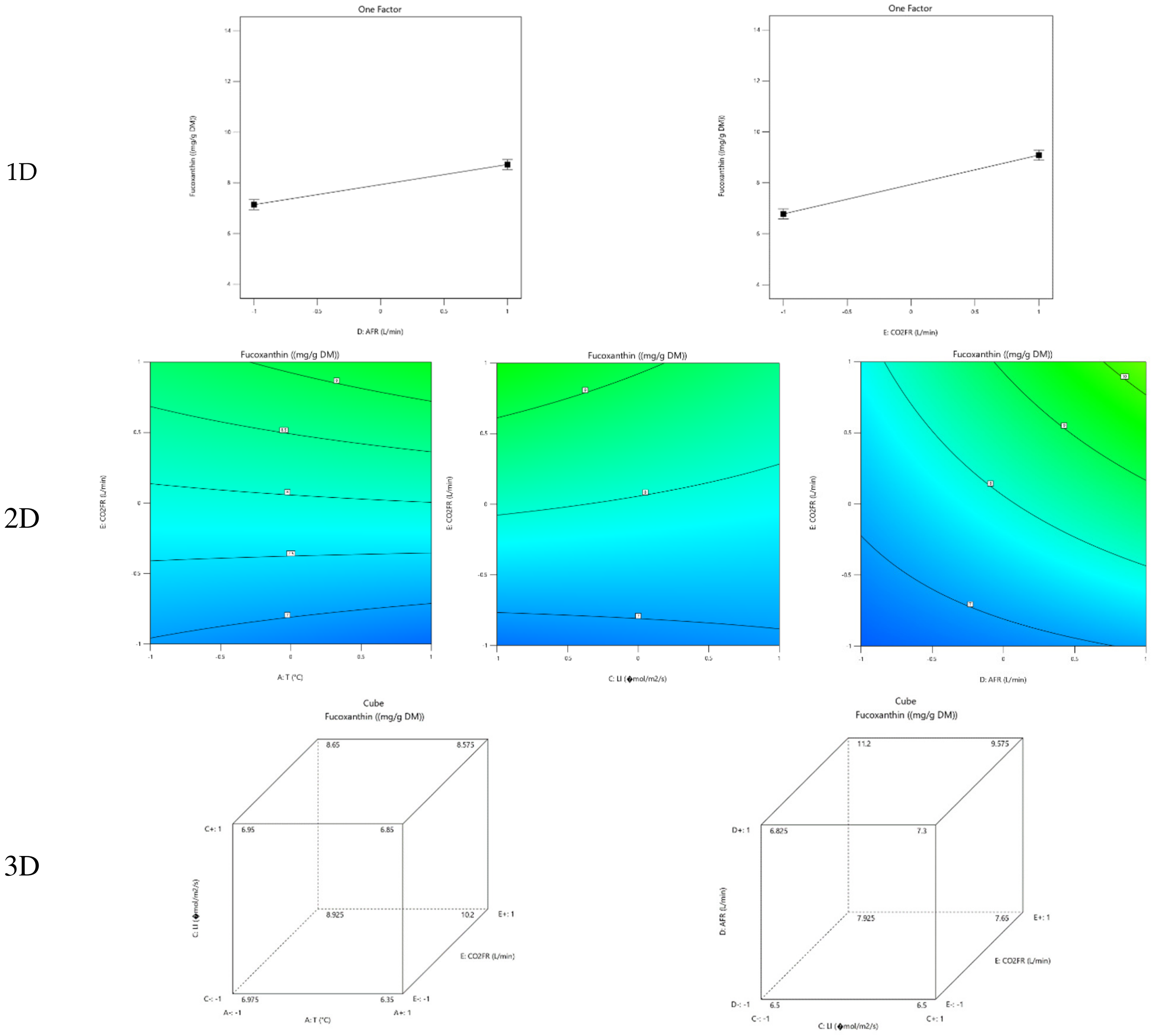

Figure S2. Illustrates the representations of fucoxanthin across one-dimensional, two-dimensional, and three-dimensional formats.

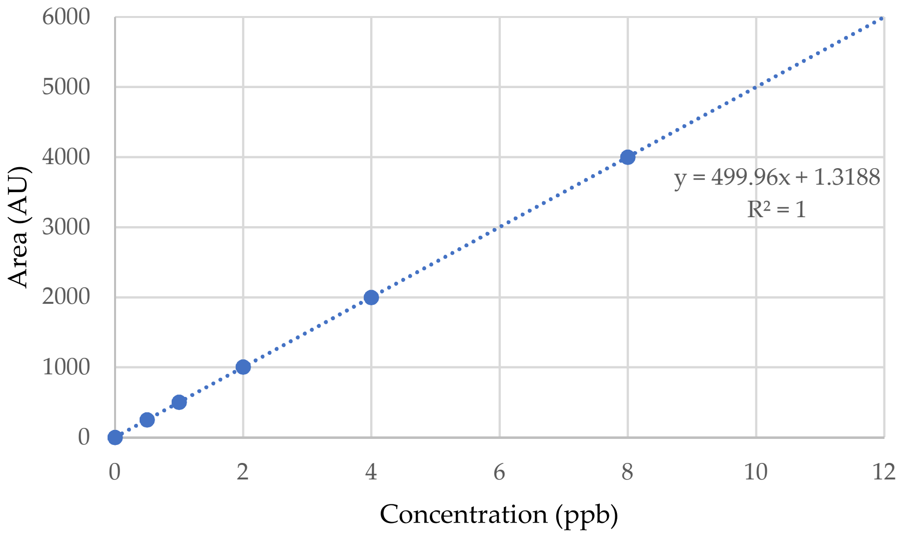

Figure S3. Calibration curve of fucoxanthin by HPLC-UV.

Table S1. Statistical parameters of the multiple regression of antioxidant activity as a function of the content of carotenoids, total phenolic compounds and fucoxanthin in cultures of the microalga *I. galbana*.

Parameters	MLR	MNLR
Equation	Z1 = 533.08+ (5.27×Z2) + (4.51×Z3) -(53.61×Z4)	Z1 = 2343.15-(272.13×Z2)-(96.80×Z3)+(293.17×Z4)+(10.49×Z2^2)+(2.63×Z3^2)-(29.94*Z4^2)
R^2	0.954	0.986
MCE	262.950	156.573
RMCE	16.216	12.513

Table S2. Fucoxanthin is used for each test in all experiments according to the design of the experiments.

Culture microalgae	Factor 1	Factor 2	Factor 3	Factor 4	Factor 5	Response
**Parameters**	**A:T**	**B:pH**	**C:LI**	**D:AFR**	**E:CO_2FR**	**Fucoxanthin**
Unit	°C		μmol/m^2/s	L/min	L/min	(mg/g)
1	-1	-1	-1	-1	-1	7.8
2	1	-1	-1	-1	-1	6.2
3	-1	1	-1	-1	-1	5.8
4	1	1	-1	-1	-1	6.2
5	-1	-1	1	-1	-1	6.7
6	1	-1	1	-1	-1	6
7	-1	1	1	-1	-1	6.5
8	1	1	1	-1	-1	6.8
9	-1	-1	-1	1	-1	6.9
10	1	-1	-1	1	-1	6.2
11	-1	1	-1	1	-1	7.4
12	1	1	-1	1	-1	6.8
13	-1	-1	1	1	-1	7.2
14	1	-1	1	1	-1	7.2
15	-1	1	1	1	-1	7.4
16	1	1	1	1	-1	7.4
17	-1	-1	-1	-1	1	7.9
18	1	-1	-1	-1	1	7.9
19	-1	1	-1	-1	1	8.1
20	1	1	-1	-1	1	7.8
21	-1	-1	1	-1	1	7.7
22	1	-1	1	-1	1	7.6
23	-1	1	1	-1	1	7.7
24	1	1	1	-1	1	7.6
25	-1	-1	-1	1	1	10.5
26	1	-1	-1	1	1	13.4
27	-1	1	-1	1	1	9.2
28	1	1	-1	1	1	11.7
29	-1	-1	1	1	1	9.7
30	1	-1	1	1	1	9
31	-1	1	1	1	1	9.5
32	1	1	1	1	1	10.1

Table S3. Coefficients of optimized models for fucoxanthin production.

Factor	Coefficient Estimate
Intercept	7.930
A-T	0.059
B-pH	-0.059
C-LI	-0.178
D-AFR	0.790
E-CO_2FR	1.150
AB	0.115
AC	-0.103
AD	0.190
AE	0.240
BC	0.178
BD	0.021
BE	-0.065
CD	-0.109
CE	-0.296
DE	0.509
ABC	0.028
ABD	-0.053
ABE	-0.078
ACD	-0.159
ACE	-0.234
ADE	0.171
BCD	0.021
BCE	0.059
BDE	-0.159
CDE	-0.228
ABCD	0.071
ABCE	0.096
ABDE	0.128
ACDE	-0.190
BCDE	0.228

Table S4. Statistical studies of the model.

**Mean**	7.930	**R^2**	0.99
**standard deviation**	0.088	**Adjusted R^2**	0.99
**Variance**	0.007	**Predicted R^2**	0.91
**Coefficient of variation**	1.11%	**Adequate precision P**	87.36%

Table S5. ANOVA for the selected factorial model.

Source	Sum of Squares	df	Mean Square	F-value	p-value	Significance
Model	90.50	30	3.02	386.15	0.04	Significant
A-T	0.11	1	0.11	14.44	0.16	Not significant
B-pH	0.11	1	0.11	14.44	0.16	Not significant
C-LI	1.02	1	1.02	129.96	0.06	Not significant
D-AFR	20.00	1	20.00	2560.36	0.01	Significant
E-CO_2FR	42.55	1	42.55	5446.44	0.01	significant
AB	0.42	1	0.42	54.76	0.08	Not significant
AC	0.34	1	0.34	43.56	0.09	Not significant
AD	1.16	1	1.16	148.84	0.06	Not significant
AE	1.85	1	1.85	237.16	0.04	Significant
BC	1.02	1	1.02	129.96	0.06	Not significant
BD	0.01	1	0.01	1.96	0.39	Not significant
BE	0.13	1	0.13	17.64	0.14	Not significant
CD	0.38	1	0.38	49.00	0.09	Not significant
CE	2.82	1	2.82	361.00	0.03	Significant
DE	8.30	1	8.30	1062.76	0.01	Significant
ABC	0.02	1	0.02	3.24	0.32	Not significant
ABD	0.09	1	0.09	11.56	0.18	Not significant
ABE	0.19	1	0.19	25.00	0.12	Not significant
ACD	0.81	1	0.81	104.04	0.06	Not significant
ACE	1.76	1	1.76	225.00	0.04	Significant
ADE	0.94	1	0.94	121.00	0.06	Not significant
BCD	0.01	1	0.01	1.96	0.39	Not significant
BCE	0.11	1	0.11	14.44	0.16	Not significant
BDE	0.81	1	0.81	104.04	0.06	Not significant
CDE	1.67	1	1.67	213.16	0.04	Significant
ABCD	0.16	1	0.16	21.16	0.13	Not significant
ABCE	0.30	1	0.30	38.44	0.10	Not significant
ABDE	0.52	1	0.52	67.24	0.07	Not significant
ACDE	1.16	1	1.16	148.84	0.06	Not significant
BCDE	1.67	1	1.67	213.16	0.04	Significant
